# Sensitivity Analysis of Excited-State Population in Plasma Based on Relative Entropy

**DOI:** 10.3390/e26090782

**Published:** 2024-09-12

**Authors:** Yosuke Shimada, Hiroshi Akatsuka

**Affiliations:** 1 Department of Electrical and Electronic Engineering, Tokyo Institute of Technology, 2-12-1-N1-10, O-Okayama, Meguro-ku, Tokyo 152-8550, Japan; 2 Kanagawa Prefectural Police Headquarters, 155-1 Yamashita-cho, Naka-ku, Yokohama, Kanagawa 231-0023, Japan; 3 Laboratory for Zero-Carbon Energy, Institute of Innovative Research, Tokyo Institute of Technology, 2-12-1-N1-10, O-Okayama, Meguro-ku, Tokyo 152-8550, Japan; hakatsuk@zc.iir.titech.ac.jp

**Keywords:** transient plasma, relative entropy, low-energy plasma, fractional Brownian motion

## Abstract

A highly versatile evaluation method is proposed for transient plasmas based on statistical physics. It would be beneficial in various industrial sectors, including semiconductors and automobiles. Our research focused on low-energy plasmas in laboratory settings, and they were assessed via our proposed method, which incorporates relative entropy and fractional Brownian motion, based on a revised collisional–radiative model. By introducing an indicator to evaluate how far a system is from its steady state, both the trend of entropy and the radiative process’ contribution to the lifetime of excited states were considered. The high statistical weight of some excited states may act as a bottleneck in the plasma’s energy relaxation throughout the system to a steady state. By deepening our understanding of how energy flows through plasmas, we anticipate potential contributions to resolving global environmental issues and fostering technological innovation in plasma-related industrial fields.

## 1. Introduction

Several methods are available for generating plasma, such as discharge ionization, thermal ionization, and photoionization, each differentiated by the manner in which energy is supplied [1]. Transient plasma, commonly observed in these generation processes, has been extensively studied in contexts such as the boundary region of nuclear fusion plasma and laser-produced plasma [2,3]. However, few methods exist for evaluating transient plasma fluctuations, particularly at low energies in a non-equilibrium state [4]. The self-consistent time-dependent CR models [5,6] where the evolution of all these quantities is calculated, including the electron energy distribution function (EEDF), have already been studied.

Recent advancements in semiconductor miniaturization necessitate a precise understanding of the fluctuation effects of electron temperature, electron density, and space potential on nanoparticle growth, as well as surface processes like etching or deposition [7]. Consequently, accurate evaluation methods for these transient plasma fluctuations are in high demand [8]. Additionally, fluctuations in the electric and magnetic fields are under investigation for devices such as the Hall thruster, a type of electric propulsion equipment [9]. Plasma also plays a crucial role in spark plugs used in automobile engines, where enhancing thermal efficiency is crucial for meeting exhaust gas regulations and improving fuel efficiency [10,11]. This necessitates a reduction in fuel consumption during combustion. Although many challenges persist in spark ignition, a novel evaluation method that considers the excitation process of atoms and molecules is needed to optimize thermal efficiency [12,13]. And welding techniques that utilize arc discharge have also been improved through the evaluation of the effect of shielding gas types [14] and the simulation method [15].

Generally, low-energy (i.e., low-temperature) plasma exhibits a low degree of ionization, requiring consideration of excitation processes due to electron collisions at various levels and collisions with ground-state atoms, along with de-excitation processes such as radiative transition. Typically, analyses of these atomic processes utilize a collisional–radiative model; however, these are predominantly conducted for plasmas in a steady state. Hence, a highly versatile evaluation method for assessing excited-state populations in transient plasma would be beneficial, particularly from the perspective of fluctuations in gas-species density, electron temperature, and electron density. Enhancing our understanding of excitation kinetics in plasmas could significantly benefit virtually every engineering sector involved in plasma applications [16].

## 2. Theoretical Backgrounds and Methods

An evaluation method for plasma fluctuations using a collisional–radiative model with the Malliavin derivative has already been demonstrated [17]. The algorithm is based on a revised argon collisional–radiative (CR) model that integrates atomic collision processes alongside conventional electron collision processes and radiative transitions.

For argon plasma, we adopted atomic data compiled by Vl$\overset{ˇ}{c}$ek for the microwave discharge argon plasma [18], and the levels involved are summarized in Table A1 in Appendix A.

The time-dependent rate equation for level *p* is provided by the following equation:

(1) $$\frac{dN_{p}}{dt}=\sum_{q\neq p}C_{qp}N_{q}N_{e}+\sum_{q\neq p}K_{qp}N_{q}N_{1}+\sum_{q>p}\Lambda_{qp}A_{qp}N_{q}+\left(\kappa_{p}N_{e}+\lambda_{p}\right)N_{i}N_{e}\;\;\;\;\;-N_{p}[\sum_{q\neq p}{(C}_{qp}+S_{p})N_{e}+\sum_{q\neq p}K_{qp}N_{1}+\sum_{q>p}\Lambda_{pq}A_{pq}],$$

where the rate coefficients are as indicated in [19]: $C_{qp}$ represents the rate coefficient of electron impact excitation or de-excitation from level *q* to *p* (details in Appendix B), $K_{qp}$ denotes the rate coefficient of ground-state atomic impact excitation or de-excitation as quenching, $\Lambda_{pq}$ denotes the optical escape factor, $A_{qp}$ denotes the rate coefficient of radiative transition probability, $\kappa_{p}$ is the rate coefficient of three-body recombination, $\lambda_{p}$ denotes the radiative recombination rate coefficient, $N_{1}$ is the ground-state atom density, $N_{e}$ denotes the electron density, and $N_{i}$ denotes the ion density.

Equation (1) is simplified into a matrix form to concurrently express other levels:

(2) $$\frac{dN}{dt}=aN+\delta ,$$

In Equation (2), $N=\left(\begin{array}{c} N_{2}\\ \cdots \\ N_{65} \end{array}\right)$ corresponds to a column of the densities of excited-level populations, $a=\left(\begin{array}{ccc} a_{2\;2} & \cdots & \cdots \\ \cdots & a_{q\;p} & \cdots \\ \cdots & \cdots & a_{65\;65} \end{array}\right)$ indicates the matrix of collisional radiative processes, of which the components are given as follows [20]:

$$a_{ji}=\left\{\begin{array}{l} \;N_{e}C_{ji}\;\;\;\;\;\;\;\;\;\;\;\;\;\;\;\;\;for\;j<i,\\ \;N_{e}C_{ji}+A_{ji}\;\;\;\;\;\;\;\;\;\;\;\;\;\;for\;j>i,\;\\ -N_{e}\left(S_{i}+\sum_{l=0,\neq i}^{M}C_{il}\right)-\sum_{l=0}^{i-1}A_{il}\;\;\;\;\;\;for\;j=i \end{array}\right.$$

and $\delta =\left(\begin{array}{c} \delta_{2}\\ \vdots \\ \delta_{65} \end{array}\right)$ corresponds to the column of the source term by a sum of electron ion recombination and excitation from the ground state, with $\delta_{j}=\alpha_{j}N_{e}^{2}N_{i}+\beta_{j}N_{e}N_{i}+C_{0j}N_{e}N_{1}$. The density of ground-state atoms was included in the analysis as an input parameter.

Brownian motion can depict stochastic phenomena such as random walks. The Wiener process satisfies the properties “Continuity”, “Mutual independence”, “Stationary increment”, and “Gaussian distribution”. Hence, fluctuations are intrinsically linked to Brownian motion, and our research has already addressed fluctuations among 64 effective excited states, alongside a ground-state level, in argon plasma. An algorithm that is specifically designed for calculating perturbations resulting from fluctuations in the density of excited states has been developed. It was confirmed that fluctuations occur due to electron collisions within argon plasma at pressures ranging from 0.1 to 10 Torr [17].

Diffusion processes such as Brownian motion are analyzed using stochastic differential equations. In this study, the atomic density at each level has a term of which the exponential part depends on time and a term expressed by the Wiener process.

When there is a fluctuation in the Wiener process at time *t*, how it affects $N_{p}(t)$ can be determined by differentiating it with respect to the Wiener process (Malliavin derivative is shown in Appendix C). We operate under the assumption that the time derivative of $N_{p}(t)$ is approximately equivalent to the Malliavin derivative $D_{t}$ of $N_{p}(t)$ [21].

To consider the impact of small changes in the trajectory of Brownian motion, we use the differentiation introduced by Malliavin, which means that when a measure shift in Wiener space is applied, only the direction in which the measure shifts is considered absolutely continuous [22,23,24].

This approach is adopted when excluding the particular solution pertaining to the steady states:

(3) $$\frac{dN_{p}(t)}{dt}\sim D_{t}N_{p}\left(t\right)=-\xi_{p}N_{p}\left(t\right),$$

In Equation (3), $\xi_{p}$ corresponds to a differential coefficient, representing the impact on the functional when a variation exists in the increment *dW* of the Wiener process. The detailed definition of the term $\xi_{p}$ will be described later in Equations (9) and (10).

To effectively evaluate the influence on the components of the collisional–radiative matrix a, the terms originating from the Malliavin derivative should be transposed from the left-hand side to the right-hand side. This repositioning allows for a clearer assessment of the effects of stochastic fluctuations on the system dynamics.

(4) 0=aN+ξN+δ,

Building on the modification of Equation (1), the number density of excited states N can be expressed using the inverse matrix of the collisional–radiative processes, which accounts for fluctuations in the electron or atom density. This formulation provides a mathematical framework to quantify the dynamic responses of the excited states to changes in the plasma environment:

(5) $$N\left(t\right)=-{a^{\prime }}^{-1}\delta ,$$

where $a^{\prime }$ consists of a sum of each matrix element of a and ξ. The time-dependent rate equation for each level is calculated from Equation (5). Furthermore, the applied calculation model assumes a one-step Wiener process for each level [8,25,26,27]. Let 0<α<1 and $\beta_{0}$ be an arbitrary real number with an initial value X(0, ω) at time 0, where $X(0,\omega )=\beta_{0}=0.$ ω designates the set of all the values of random (ω belongs to a sample space Ω). For t>0, fractional Brownian motion $X\left(t,\omega \right)$ is defined as follows:

(6) $$X\left(t,\omega \right)=\frac{1}{\Gamma (\alpha +\frac{1}{2})}\left[\int_{-\infty }^{0}\left\{{\left(t-s\right)}^{\alpha -\frac{1}{2}}-{\left(-s\right)}^{\alpha -\frac{1}{2}}\right\}dX\left(s,\omega \right)+\int_{0}^{t}{\left(t-s\right)}^{\alpha -\frac{1}{2}}dX\left(s,\omega \right)\right].$$

Simplifying the notation, we denote $X\left(t,\omega \right)$ as $X\left(t\right)$. According to this definition, the increments of fractional Brownian motion, $X\left(t\right)$, are correlated, except when $\alpha =\frac{1}{2}$; in this case, the increments are independent of each other. Equation (7) is non-zero for $\alpha \neq \frac{1}{2}$.

(7) $$Ε\left(X\left(t\right)(X\left(t+h\right)-X\left(t\right))\right)=\frac{1}{2}\left[{\left(t+h\right)}^{2\alpha }-t^{2\alpha }-h^{2\alpha }\right]$$

When $\alpha >\frac{1}{2}$, the sign of $X\left(t\right)-X\left(0\right)$ and that of $X\left(t+h\right)-X(t)$ are frequently aligned, indicating persistence in the direction of movement. Conversely, when $\alpha <\frac{1}{2}$, these signs often become antiparallel, suggesting a tendency toward reversals in direction.

Applying the properties of fractional Brownian motion to the Malliavin derivative, the parameter $\xi_{p}$ in Equation (3) is modified to $\xi_{p}^{2\alpha }$:

(8) $$D_{t}N_{p}\left(t\right)=-\xi_{p}^{2\alpha }N_{p}\left(t\right),$$

where the range of α is [0, 1]. In this paper, FRC that is an abbreviation of fractal, and the same meaning of the Hurst exponent is defined as 2α.

For example, to treat fluctuation by electron collision, the term of $\xi_{p}^{2\alpha }$ is calculated as

(9) $$\xi_{p}^{2\alpha }=\sum_{n\neq p}\left({-C}_{p,n}^{2\alpha }+C_{n,p}^{2\alpha }\right).$$

Therefore, when calculating the fluctuation of atom density of all the excited states, the terms $\sum_{n\neq p}\left({-C}_{p,n}^{2\alpha }+C_{n,p}^{2\alpha }\right)$ should be added to the right-hand side of Equation (1). On the other hand, for another case of fluctuation, i.e., by ground-state atomic collisions, the term of $\xi_{p}^{2\alpha }$ becomes

(10) $$\xi_{p}^{2\alpha }=\sum_{n\neq p}\left({-K}_{p,n}^{2\alpha }+K_{n,p}^{2\alpha }\right).$$

The analysis of fluctuations resulting from electron collisions and ground-state atom collisions was conducted using the Malliavin derivative. However, except for states experiencing large fluctuations, the atomic density significantly varied across different levels, complicating the detection of the time constant for these fluctuations [15].

For effective spectroscopic analysis or control of a plasma system, confirming state fluctuations at each level is desirable. Additionally, optimizing plasma excitation processes from the perspective of energy efficiency is beneficial. This optimization can be achieved by applying the concept of entropy to assess the variation in the atomic number density of each state relative to others, facilitating more efficient energy usage and process control.

By employing the relative entropy method [28] that analyzes non-adiabatic entropy, we study transient plasma behavior through the collisional–radiative model. The relative entropy $\sigma_{na}$ is formulated using Equation (11), which involves the time derivative of the Kullback–Leibler divergence, denoted as D [29]. In this context, $k_{B}$ represents the Boltzmann constant, $P\left(t\right)$ is the distribution function at time *t*, and $P^{SS}\left(\tau_{t}\right)$ is the distribution function in the steady state. $\tau_{t}$ is the control parameter.

(11) $$\sigma_{na}=-k_{B}\dot{D}\left(P\left(t\right)∥P^{SS}\left(\tau_{t}\right)\right),$$

Kullback–Leibler information is defined as

(12) $$D\left(P\left(t\right)∥P^{SS}\left(\tau_{t}\right)\right)\equiv \sum_{x}P(x,t)\ln\frac{P(x,t)}{P^{SS}(x,\tau_{t})}\;\;\;\;\;\;\;\;\;\;\;\;\;\;\;\;\;\;=\sum_{x}P(x,t)\ln P(x,t)-\sum_{x}P\left(x,t\right)\ln P^{SS}\left(x,\tau_{t}\right)$$

which represents a kind of distance between the two distributions.

The time variation matrix of each minute time in Equation (12) is symbolized as $\hat{T}$, which is expressed as

(13) $$\hat{T}=e^{W(\tau_{t})∆t}.$$

$W(\tau_{t})$ is a state transition matrix by heat exchange with a heat bath (in this paper, this means ionic atoms and ground-state atoms) corresponds to the product of the inverse matrix of a and row of the source term δ by a sum of electron ion recombination and excitation from the ground state.

The time variation in Kullback–Leibler information is expressed as

(14) $$\dot{D}\left(P\left(t\right)∥P^{SS}\left(\tau \right)\right)\equiv \underset{∆t\to 0}{\lim}\frac{1}{∆t}\left[D\left(\hat{T}P\left(t\right)∥{\hat{T}P}^{SS}\left(\tau \right)\right)-\left(P\left(t\right)∥P^{SS}\left(\tau \right)\right)\right],$$

when the time variation in the steady state is exceedingly small compared with that in the transient state:

(15) $${\hat{T}P}^{SS}\left(\tau \right)=P^{SS}\left(\tau \right).$$

Hence, the time rate of change of Kullback–Leibler information is expressed as (from Appendix D)

(16) $$-\dot{D}\left(P\left(t\right)∥P^{SS}\left(\tau \right)\right)=\sum_{x,x^{\prime }}W_{x,x\prime }P(x^{\prime },t)\ln\frac{P^{SS}(x,\tau )P(x\prime ,t)}{P(x,t)P^{SS}(x\prime ,\tau )}.$$

The time variation matrix of each time step of the Wiener process is symbolized as $\hat{U}$, which is expressed as

(17) $$\hat{U}=e^{W^{\prime }(\tau_{t})∆W}.$$

Suppose P(x,t) and P(x′,t) in Equation (16) are generated by $\hat{U}$:

$W^{\prime }(\tau_{t})$ is also a state transition matrix, which, by heat exchange with a heat bath, corresponds to the product of the inverse matrix of a′ and row of the source term δ.

(18) $$P\left(t\right)={\hat{U}P}^{SS}\left(\tau \right).$$

The argument of the natural logarithm in the non-adiabatic entropy formula can be regarded as the weight of each transition rate by heat exchange with a heat bath. And in the case that the requirement of detailed balancing are met:

(19) $$W_{x,x\prime }P^{SS}\left(x\prime \right)={W_{x\prime ,x}P}^{SS}\left(x\right),$$

where this steady state is also seen as an equilibrium state.

Valuable information for analyzing the sensitivity of each plasma level in a transient state is obtained by evaluating the time variation of this weight at each level, achieved by considering the ratio of the distribution function of the transient plasma (when changing the FRC) to the distribution function in the steady state. In this report, to evaluate the magnitude of local fluctuations, we set x′ as x+1 for each state. This analysis was particularly useful for examining the fluctuations of macroscopic plasma parameters, such as $T_{e}$ or $N_{e}$, through the kinetics of the excited-state population. From these results, the sensitivity to fluctuations can be calculated in the population density of excited states caused by electron collisions or atomic collisions at each level.

## 3. Results

The numerical procedure outlined above was employed to evaluate the sensitivity of transient plasma fluctuations through each elementary process, such as electron collisions. According to reference [30], plasmas are categorized by parameters such as plasma density and electron temperature. Our focus is on low-energy plasmas generated in laboratory-scale experiments. Consequently, the glow discharge, arc discharge, and recombining afterglow plasma were assessed via the method proposed in this paper.

In this study, we specifically calculated the fluctuation of the excited-state density of Ar atoms. The primary kinetic processes considered are electron collisions and ground-state atomic collisions. The fluctuations of each excited-state atom are evaluated in terms of transitions from each level to all other levels, as the transition from one level to another is significant, particularly at high-energy states. Therefore, we analyzed the density fluctuations from each level (*p* = 2–65) to all upper and lower levels, capturing the comprehensive dynamics of state transitions within the plasma.

### 3.1. Glow Discharge

Today, glow discharge plasma is utilized as a light source in devices such as a neon sign [31]. Additionally, this type of plasma is applied in analytical chemistry, where the emitted light is spectroscopically analyzed to glean information regarding atomic interactions within the gas. In the context of a DC discharge tube, when the current is increased beyond a Townsend discharge, the system is characterized by the space charge effect, the diffusion of charged particles into the wall, and the emission of secondary electrons [32]. This plasma is an example of ionizing plasma, and excitation from ground-state atoms and multi-step excitation are characteristic of the corona phase under Griem’s boundary and ladder-like excitation–ionization above the boundary.

**Figure 1:**
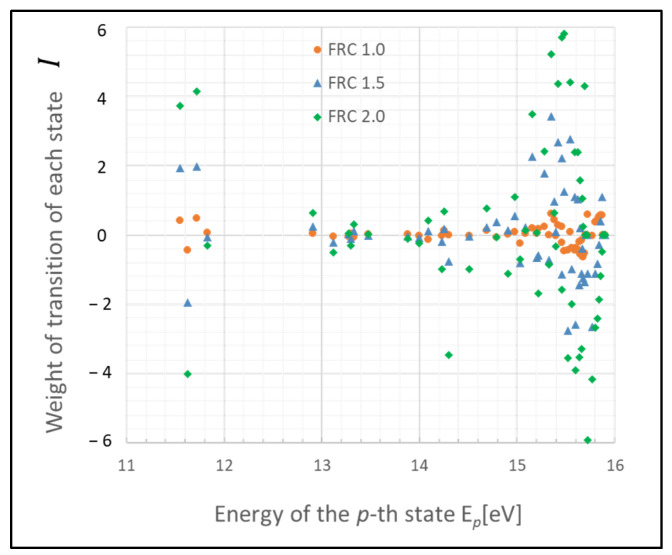
Plots of non-adiabatic entropy weights in glow discharge plasma, calculated for each level using the revised model influenced by electron collisions. Dependency on the parameter FRC is illustrated (conditions: 1 Torr, 500 K, 1 eV, and 10^9^ cm^−3^).

The graph in Figure 1 illustrates a scenario where the gas pressure is 1 Torr, the gas temperature *T_g_* is 500 K, the electron temperature *T_e_* is 1 eV, and electron density *N_e_* is 10^9^ cm^−3^, with FRC indicating 2α. The weight of each transition rate is defined as Ι(x,x′)

(20) $$Ι\left(x,x^{\prime }\right)\equiv \ln\frac{P^{SS}\left(x,\tau \right)P\left(x^{\prime },t\right)}{P\left(x,t\right)P^{SS}\left(x^{\prime },\tau \right)}.$$

The weight of each transition exhibits varying levels of fluctuation in each state, with deviations from zero likely indicating the extent of influence of the Wiener process. Notably, atoms in states p= 2–4 (11.548–11.723 eV) and p>20 (14.509 eV) demonstrate substantial fluctuations based on FRC. This outcome is largely due to the infrequent radiative transitions, rendering these states predominantly influenced by collisional processes, which allow for atoms to survive longer. The discrepancy in the number density of excited-state atoms is particularly significant between states p= 2–4.

**Figure 2:**
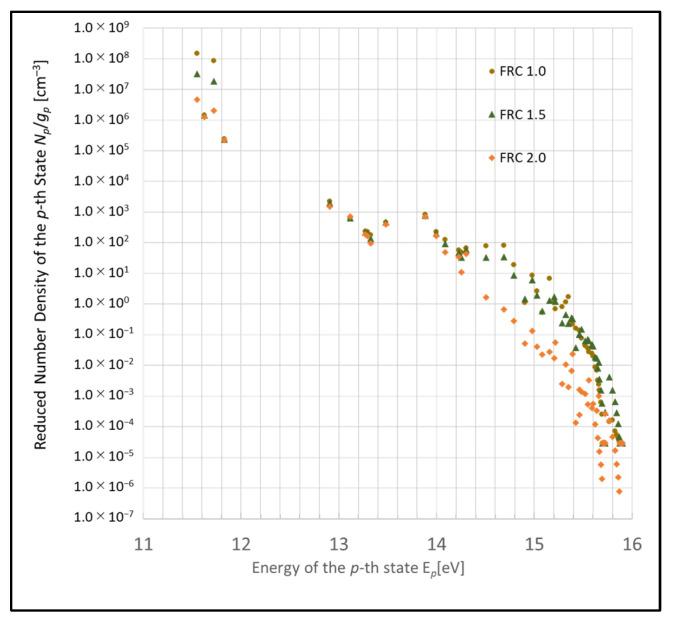
Boltzmann plots illustrating the revised model due to electron collision and their dependency on the FRC parameter (conditions: 1 Torr, 500 K, 1 eV, and 10^9^ cm^−3^).

Furthermore, a substantial difference is noted in the tendency of fluctuations (i.e., increase or decrease) between the state p>20 and p<20 compared with that in Figure 2. This difference arises because the energy gaps in higher-energy states are smaller compared with those in lower-energy states.

**Figure 3:**
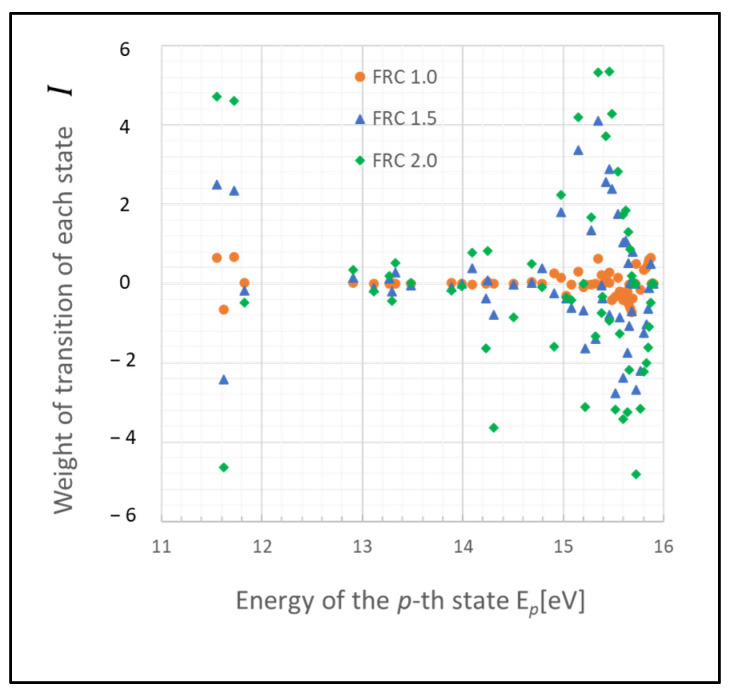
Plots of non-adiabatic entropy weights in glow discharge plasma, calculated for each level using the revised model, influenced by electron collisions. Dependency on the parameter FRC is shown (conditions: 0.1 Torr, 500 K, 1 eV, and 10^9^ cm^−3^).

**Figure 4:**
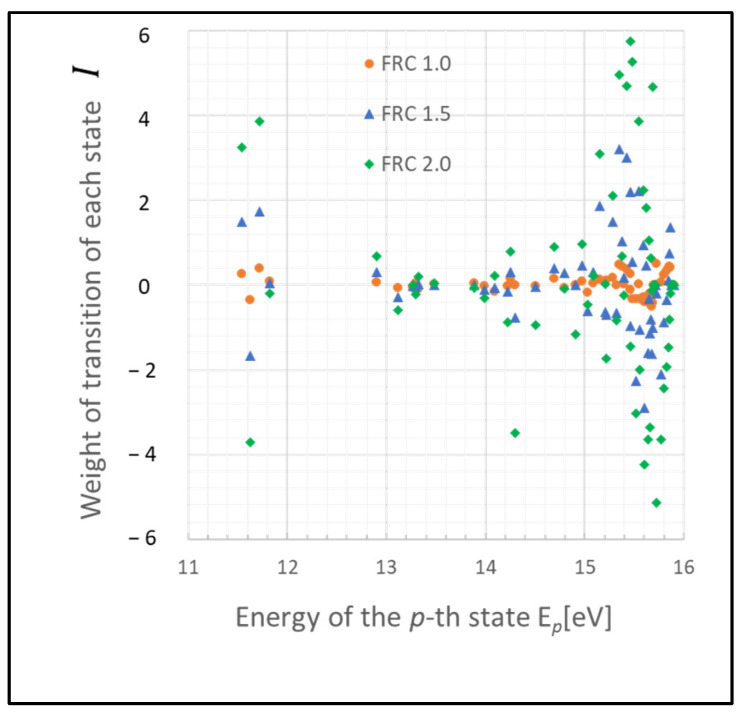
Plots of non-adiabatic entropy weights in glow discharge plasma, calculated for each level using the revised model due to electron collisions. The dependency on the parameter FRC is depicted (conditions: 1 Torr, 1000 K, 1 eV, and 10^9^ cm^−3^).

These states may function as a bottleneck in relaxing the entire plasma system to the steady state, as inferred from the relative entropy of the state. The graph in Figure 3 depicts the weight of the relative entropy for a case where the gas pressure is 0.1 Torr, with other parameters being the same as those shown in Figure 1. Comparing Figure 1 and Figure 3, an increase in the pressure of the discharge gas is observed to accelerate the convergence of non-adiabatic entropy. Similarly, Figure 4 represents another scenario where the gas temperature is 1000 K, with other parameters consistent with Figure 1. A comparison between the cases of Figure 1 and Figure 4 shows that an increase in the temperature of the discharge gas also slightly accelerates the convergence of non-adiabatic entropy.

### 3.2. Arc Discharge

Arc discharge is extensively utilized in applications such as welding and arc lamps, and research into its utility in developing electric-propulsion spacecraft is ongoing. In a DC discharge tube, arc discharge occurs when the current is increased beyond that used for glow discharge. In contrast to nuclear fusion plasma, field emission has a weak contribution because its penetration gap is small and the density of volume electrons is high, and the discharge voltage is typically lower than that observed in glow discharge [33,34]. Compared to glow discharge, excitation and de-excitation due to electron collisions from adjacent levels are dominant, the slight contribution of far-away states makes the difference of Ι(x,x′) each state small, and the system is close to the thermal equilibrium from Equation (19).

**Figure 5:**
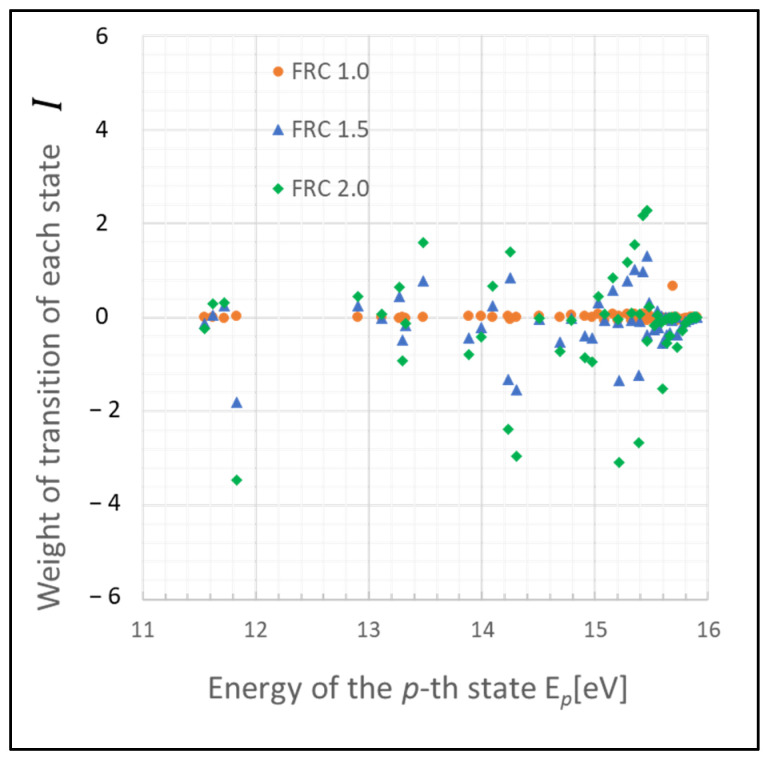
Plots of non-adiabatic entropy weights in arc discharge plasma, calculated for each level using the revised model due to electron collisions. The dependency on the parameter FRC is demonstrated (conditions: 760 Torr, 500 K, 2 eV, and 10^17^ cm^−3^).

The graph in Figure 5 illustrates a scenario where the gas pressure is 760 Torr, the gas temperature is 500 K, the electron temperature is 2 eV, and the electron density is 10^17^ cm^−3^, with FRC indicating 2α. The results from Figure 5 show that the convergence of states in arc discharge is faster than that observed in glow discharge, with the weights in the transition to a steady state being more uniformly distributed across all states. This indicates that the processes of electron collision and ground-state atomic collision occur at a relatively higher frequency in arc discharge compared with glow discharge, thereby reducing the time duration between transient and steady-state plasma.

Particularly, in the case of CO_2_ laser weld of steel sheets, the influence of shielding gas types and flow rates have already studied [35]. For example, the tensile strength and formability of laser welds were studied, and they are strongly dependent upon the shielding gas types. Moreover, the shielding gas speed affects the weld width. In this plasma, it was reported that the fluctuation was caused and heat loss also produced. Our proposed method may be related to cases like this.

### 3.3. Recombining Plasma

In laboratory discharges, the cessation of the discharge process leads to the diffusion and recombination of charged species into neutral species, forming what is known as afterglow plasma [36]. This plasma also serves as a source of radicals generated from specialized discharges, which are transported to a chamber through a dedicated transport tube, a process referred to as “Remote Plasma” [37], where excited-state atoms are originated from the electron-ion recombination, rather than electron-impact excitation, since the electrons do not have sufficient energy for excitation.

**Figure 6:**
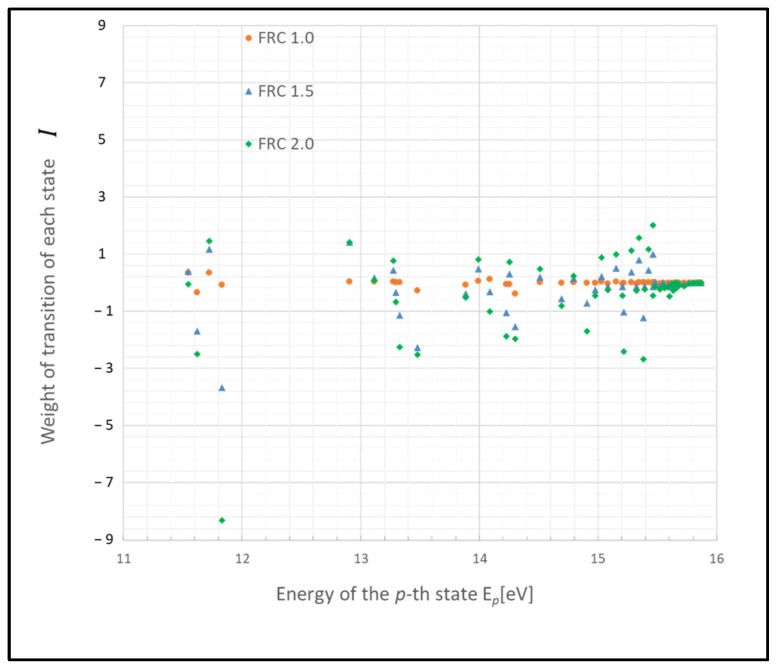
Plots of non-adiabatic entropy weights in recombining plasma, calculated for each level using the revised model influenced by electron collisions. The dependency on the parameter FRC is illustrated (conditions: 0.0075 Torr, 500 K, 0.2 eV, and 10^13^ cm^−3^).

**Figure 7:**
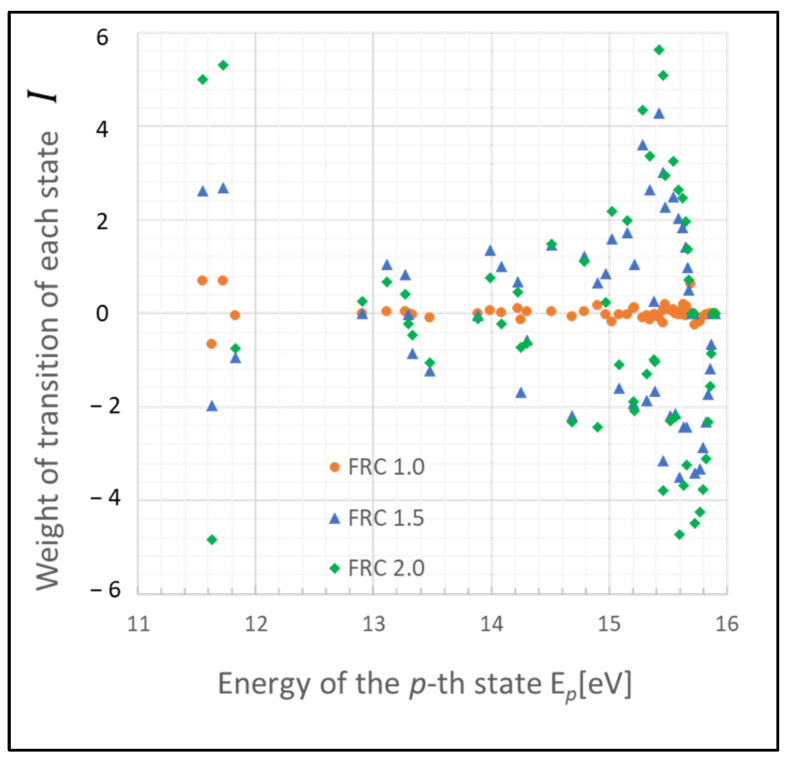
Plots of non-adiabatic entropy weights in recombining plasma, calculated for each level using the revised model, influenced by electron collisions. Dependency on the parameter FRC is shown (conditions: 0.0075 Torr, 500 K, 0.2 eV, and 10^9^ cm^−3^).

**Figure 8:**
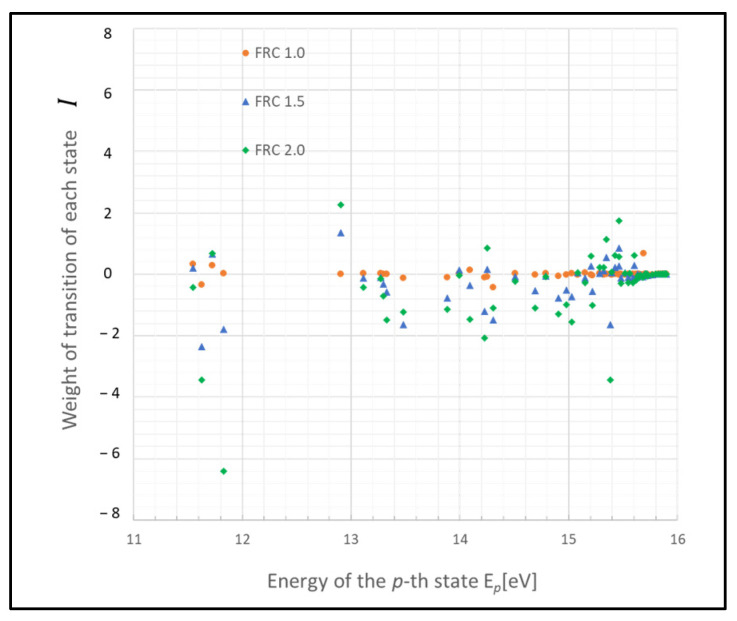
Plots of non-adiabatic entropy weights in recombining plasma, calculated for each level using the revised model influenced by electron collisions. The plots illustrate the dependency on the parameter FRC (conditions: 0.0075 Torr, 500 K, 0.1 eV, and 10^13^ cm^−3^).

The graphs in Figure 6, Figure 7 and Figure 8 depict scenarios with a gas pressure of 0.0075 Torr and a temperature of 500 K, with FRC indicating 2α. Figure 6 shows an electron density of 10^13^ cm^−3^ and an electron temperature of 0.2 eV. Figure 7 shows an electron density of 10^9^ cm^−3^ and the same electron temperature of 0.2 eV. A comparison between the results of Figure 6 and Figure 7 reveals that as the electron density decreases, the states with high weights of relative entropy predominantly include lower-energy states (metastable states) and relatively high-energy states. Particularly, in high-energy states, an increase is noted in the high weight of relative entropy corresponding to the increase in energy due to energy flow dynamics.

Figure 8 shows that although the electron density remains at 10^13^ cm^−3^, the electron temperature decreases to 0.1 eV, indicating a general decrease in the weight of relative entropy across almost all states compared with Figure 6. This indicates that lower electron temperatures lead to a decrease in the relative entropy across plasma states.

The graph in Figure 6 illustrates an increase in the dominance of relative entropy from around the middle of the states (about 14.5 eV) of the system, extending to both upper and lower states, suggesting two distinct energy flows originating from around the middle levels.

## 4. Discussion

Generally, when energy is transferred, fluctuation of entropy tends to be higher on the supply side compared with the demand side. Additionally, relative fluctuation of entropy is typically greater on the demand side than on the supply side [38].

(21) $$\frac{{\langle S_{-}^{2}\rangle }_{c}/\tau }{{\left[\langle S_{-}\rangle /\tau \right]}^{2}}\geq \frac{{\langle S_{+}^{2}\rangle }_{c}/\tau }{{\left[\langle S_{+}\rangle /\tau \right]}^{2}}$$

$S_{+}$ denotes entropy generated in a low-temperature bath, $S_{-}$ denotes entropy generated in a high-temperature bath, and τ denotes time that is longer than a time step of the Wiener process. From Equation (21), the absolute value of the weight of each transition rate increases from the energy supply side to the energy demand side. The subscript c of the entropy indicates cumulant and $\langle x^{n}\rangle \equiv \sum_{x}x^{n}P(x)$ indicates moment. For example, ${\langle x^{2}\rangle }_{c}=\langle {\left(x-\langle x\rangle \right)}^{2}\rangle$.

However, in systems such as plasmas where radiative transitions serve as energy dissipation pathways, considering both the trend of relative fluctuation of entropy and the radiative process’s contribution to the lifetime is crucial [39]. In the report, statistical fluctuations in a steady-state system in a thermal equilibrium are influenced by external perturbations. They set response function as R(t,s), an impulse changing the potential $U\to U-h_{S}V$ at time s, in a quantity Q at time t≥s,

(22) $$R_{QV}\left(t,s\right)=\frac{\beta }{2}\frac{d}{ds}\langle V\left(s\right)Q(t)\rangle -\frac{\beta }{2}\langle \frac{d}{ds}V\left(s\right)Q(t)\rangle$$

From our results, the trend of relative fluctuation of entropy corresponds to the first term, and the lifetime of radiative processes corresponds to the second one.

For general thermodynamic systems described as Markov processes, a universal trade-off relation between efficiency and power is established [40]. The findings from plasma studies suggest that levels exhibiting high weights of relative entropy may be critical in facilitating the relaxation of the entire plasma system to a steady state. Controlling these levels selectively from the outside is essential for optimization, particularly from the perspective of energy efficiency.

In scenarios where the corona model does not apply, and cumulative excitation or de-excitation dominates in relatively high electron density environments, traditional methods such as the modified Boltzmann plot in the corona-phase region [41,42,43] may not be suitable [44]. Nevertheless, our evaluation method effectively reveals the direction and strength of these physical tendencies via calculation of the weight of each transition rate *I* defined in Equation (20).

**Figure 9:**
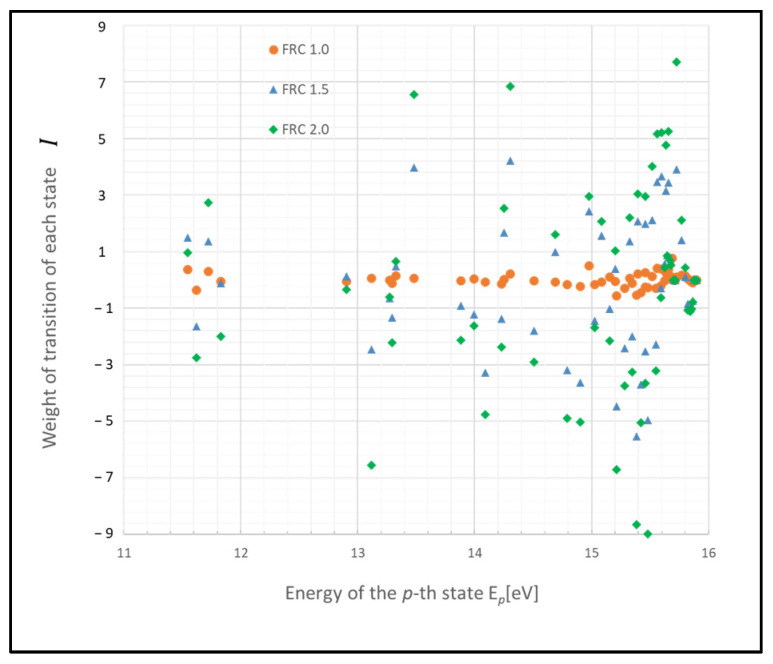
Plots of non-adiabatic entropy weights in glow discharge plasma, calculated using the revised model for each level affected by collisions with ground-state atoms. The dependency on the parameter FRC is shown (conditions: 1 Torr, 500 K, 1 eV, and 10^9^ cm^−3^).

In actual experimental situations involving glow discharge, fluctuations caused by ground-state atoms also occur, as demonstrated in Figure 9. Compared with fluctuations induced by electron collisions, a similar degree of weight across all levels is observed. This indicates that in the energy transport due to electron collisions, the weight on the transition to a steady state tends to be evened out across the system by collisions involving ground-state atoms. However, to simultaneously evaluate fluctuations due to electron collisions and fluctuations due to the ground-state atoms, it is thought that the contributions cannot be simply combined. Because when a measure in Wiener space shifts, only the direction in which the absolutely continuous measure shifts is considered, and the shift of the measure also changes the basis of another shift by $de_{i}$.

(23) $$de_{i}=\Gamma_{jk}^{i}{d\xi }^{j}e_{k}$$

$\Gamma_{jk}^{i}$ denotes the connection of vector from one point to another one. Hence, using a tensor is necessary for the combination of two shifts. Particularly, depending on the order of each shift in the Wiener space, the difference of the combination of shifts is presumably to be evaluated by the product of the torsion tensor $S_{jk}^{\;i}$ expressed in Equation (24) and the infinitesimal area $f^{jk}$ spanned by these shifts [45,46].

(24) $$S_{jk}^{i}=\frac{1}{2}\left(\Gamma_{jk}^{i}-\Gamma_{kj}^{i}\right)$$

In contrast, since the number of atoms at each level is always a positive value, a realistic method is to analyze the effects of electron collisions and ground-level atomic collisions alternately using the exponential gradient method with a non-negative matrix [47,48].

Overall, the results display a symmetrical tendency about the origin for each level and its adjacent levels. This symmetry suggests that when the stochastic flow locally between each level is considered, it is almost antisymmetric with respect to the exchange of states. Therefore, the positive and negative values in Equation (20) are relative. However, in complex physical models, the stationary distribution may be multi-modal due to nonlinear dependencies between parameters or latent (or hidden) variables [49].

For other plasmas, such as those naturally occurring or in fusion reactors, where plasma density and temperature differ from the studied cases, assessing the applicability of the CR model used in the above analysis is necessary. Despite limitations, this method could potentially be adapted for these plasmas by refining it from the perspective of entropy.

We did not evaluate plasma during the cooling process, where the EEDF shows strong departure from the Maxwell distribution. The self-consistent description of the EEDF and the population density remains as a future task.

## 5. Conclusions

An evaluation method is proposed to utilize relative entropy to predict plasma fluctuations based on a revised collisional–radiative model and fractional Brownian motion. Statistical analyses conducted via this method revealed that the transient behavior of plasma is characterized by both small and large fluctuations in each state. Information (i.e., *I*) is proposed as indicator to evaluate how far a system is from its steady state. These findings highlight the necessity of considering both the trend of entropy and the contribution of the radiative process to the lifetime. Additionally, states exhibiting high relative entropy weights may be critical in facilitating the relaxation of plasma to a steady state throughout the entire system.

## Data Availability

The original contributions presented in the study are included in the article, further inquiries can be directed to the corresponding author.

## Appendix A

The levels involved in the discharge argon plasma are summarized in Table A1 below.

**Table A1:** Excited levels of Ar I considered in the present Ar CR model [18,50].

Level Number *p*	Designation $n_{pqn}l\;{\left[K\right]}_{J}$	Excitation Energy $E_{p}$ (eV)	Statistical Weight $g_{p}$	Level Number *p*	Designation $n_{pqn}l\;{\left[K\right]}_{J}$	Excitation Energy $E_{p}$ (eV)	Statistical Weight $g_{p}$
1	3p^6^	0.000	1	35	6f, g, h	15.382	216
2	4s[3/2]_2_	11.548	5	36	8p′	15.600	12
3	4s[3/2]_1_	11.624	3	37	8p	15.423	24
4	4s′[1/2]_0_	11.723	1	38	7d′ + 9s′	15.636	24
5	4s′[1/2]_1_	11.828	3	39	7d + 9s	15.460	48
6	4p[1/2]_1_	12.907	3	40	7f′, g′, h′, i′	15.659	160
7	4p[3/2]_1,2_, [5/2]_2,3_	13.116	20	41	7f, g, h, i	15.481	320
8	4p′[3/2]_1,2_	13.295	8	42	8d′, f′, ⋯	15.725	240
9	4p′[1/2]_1_	13.328	3	43	8d, f, ⋯	15.548	480
10	4p[1/2]_0_	13.273	1	44	9p′, d′, f′, ⋯	15.769	320
11	4p′[1/2]_0_	13.480	1	45	9p, d, f, ⋯	15.592	640
12	3d[1/2]_0,1_, [3/2]_2_	13.884	9	46	10′	15.801	400
13	3d[7/2]_3,4_	13.994	16	47	10	15.624	800
14	3d′[3/2]_2_, [5/2]_2,3_	14.229	17	48	11′	15.825	484
15	5s′	14.252	4	49	11	15.648	968
16	3d[3/2]_1_, [5/2]_2,3_ + 5s	14.090	23	50	12′	15.843	576
17	3d′[3/2]_1_	14.304	3	51	12	15.666	1152
18	5p	14.509	24	52	13′	15.857	676
19	5p′	14.690	12	53	13	15.680	1352
20	4d + 6s	14.792	48	54	14′	15.868	784
21	4d′ + gs′	14.976	24	55	14	15.691	1568
22	4f′	15.083	28	56	15′	15.877	900
23	4f	14.906	56	57	15	15.700	1800
24	6p′	15.205	12	58	16′	15.884	1024
25	6p	15.028	24	59	16	15.707	2048
26	5d′ + 7s′	15.324	24	60	17′	15.890	1156
27	5d + 7s	15.153	48	61	17	15.713	2312
28	5f′, g′	15.393	64	62	18′	15.895	1296
29	5f, g	15.215	128	63	18	15.718	2592
30	7p′	15.461	12	64	19′	15.899	1444
31	7p	15.282	24	65	19	15.722	2888
32	6d′ + 8s′	15.520	24				
33	6d + 8s	15.347	48				
34	6f′, g′, h′	15.560	108				

## Appendix B

In this study, the EEDF is assumed to be Maxwellian, following Fujimoto [19]. We admit that it will be necessary to solve the Boltzmann equation with the reduced electric field as the input in more detail. However, this is a future task and out of scope in the present study. First of all, this study considered the excited-state density distribution from an entropic standpoint, and in that sense, it is sufficient to discuss it under the assumption of a Maxwellian distribution. The excitation rate coefficient for Ar is provided as

(A1) $$C\left(p,q\right)=\sqrt{\frac{2}{m_{e}}}\int_{\varepsilon_{th}}^{\infty }\sqrt{\varepsilon }\sigma_{p,q}\left(\varepsilon \right)F\left(\varepsilon \right)d\varepsilon .$$

In Equation (A1), $m_{e}$ represents the mass of the electron, and $\varepsilon_{th}$ represents the threshold energy of the process. The double-subscript notation p and q in the electron collisional excitation cross-section $\sigma_{p,q}\left(\varepsilon \right)$ denote the initial and final states of atoms, respectively (effective principal quantum number), following Vlček’s paper [18]. $F\left(\varepsilon \right)$ is the electron energy distribution function (EEDF), which is assumed to be Maxwellian in this study, which is formulated with normalization, $\int f\left(\varepsilon \right)d\varepsilon =1$, as below:

(A2) $$F\left(\varepsilon \right)=2\sqrt{\frac{\varepsilon }{\pi }}\cdot {\left(\frac{1}{kT_{e}}\right)}^{\frac{3}{2}}\exp\left(-\frac{\varepsilon }{kT_{e}}\right).$$

From Equation (A2), the average energy is $\bar{\varepsilon }=\frac{3k_{B}T_{e}}{2}.$ Therefore, these numbers do not represent the principal quantum number. $\sigma_{p,q}\left(\varepsilon \right)$ is determined based on the transition as described by an optically allowed transition

(A3) $$\sigma_{p,q}^{A}\left(\varepsilon \right)=4\pi a_{0}^{2}{\left(\frac{\varepsilon_{1}^{H}}{\varepsilon_{p,q}}\right)}^{2}f_{p,q}\psi_{p,q}^{A}U_{p,q}^{-1}\left(1-U_{p,q}^{-1}\right)\ln\left(1.25\omega_{p,q}U_{p,q}\right)$$

parity forbidden transitions

(A4) $$\sigma_{p,q}^{P}\left(\varepsilon \right)=4\pi a_{0}^{2}\psi_{p,q}^{A}U_{p,q}^{-1}\left(1-U_{p,q}^{-1}\right)$$

and spin forbidden transitions

(A5) $$\sigma_{p,q}^{S}\left(\varepsilon \right)=4\pi a_{0}^{2}\psi_{p,q}^{S}U_{p,q}^{-3}\left(1-U_{p,q}^{-2}\right)$$

In each equation, $a_{0}$ denotes the Bohr radius, $\varepsilon_{1}^{H}$ denotes the ionization energy of hydrogen atoms, Up,q=εεp,q(${\varepsilon >\varepsilon }_{p,q}$) denotes the normalized dimensionless energy, $\psi_{p,q}$ and $\omega_{p,q}$ are the fitting parameters, and $f_{p,q}$ denotes the oscillator strength for the case of an optically allowed transition.

## Appendix C

Malliavin analysis evaluated the increase in Brownian motion. Brownian motion B(t) is expressed below. The increase in it is set as $\Delta_{i}=B\left(t_{i+1}\right)-B\left(t_{i}\right),(i=0,1,\cdots n-1)$.

(A6) $$B\left(t\right)=\left\{B\left(t\right)-B\left(t_{n-1}\right)\right\}+\left\{B\left(t_{n-1}\right)-B\left(t_{n-2}\right)\right\}+\cdots +\left\{B\left(t_{1}\right)-B\left(t_{0}\right)\right\}$$

And the stochastic process is expressed below.

(A7) $$S(t)=f_{n}(\Delta_{0},\Delta_{1},\cdots ,\Delta_{n-1})$$

In this case, the Malliavin derivative $D_{t}$ is defined below. The maximum division of time set as Π.

(A8) $$D_{t}S(t)=\underset{\left|\Pi \right|\to 0}{\lim}\frac{\partial f_{n}}{\partial {∆}_{j}}(\Delta_{0},\Delta_{1},\cdots ,\Delta_{n-1})$$

## Appendix D

The time variation of Kullback–Leibler information is expressed below.

(A9) $$\dot{D}\left(P\left(t\right)∥P^{SS}\left(\tau \right)\right)\equiv \underset{∆t\to 0}{\lim}\frac{1}{∆t}\left[D\left(\hat{T}P\left(t\right)∥{\hat{T}P}^{SS}\left(\tau \right)\right)-\left(P\left(t\right)∥P^{SS}\left(\tau \right)\right)\right]\;\;\;\;=\sum_{x}d_{t}P\left(x,t\right)(\ln P\left(x,t\right)-\ln P^{SS}\left(x,\tau \right))\;\;\;\;=-\sum_{x,x^{\prime }}W_{x,x\prime }P(x^{\prime },t)\ln\frac{P^{SS}(x,\tau )P(x\prime ,t)}{P(x,t)P^{SS}(x\prime ,\tau )}$$
